# Successful transcatheter pulmonary valve implantation under elective extracorporeal membrane oxygenation in a patient with tetralogy of Fallot with biventricular failure and ventricular tachycardia: a case report

**DOI:** 10.1093/ehjcr/ytag044

**Published:** 2026-01-29

**Authors:** Hidehiro Mori, Mizuhiko Ishigaki, Sung-Hae Kim, Akane Shibuya, Yasuhiko Tanaka

**Affiliations:** Department of Cardiology, Shizuoka Children’s Hospital, 860, Urushiyama, Aoi-ku, Shizuoka, 420-8660, Japan; Department of Cardiology, Shizuoka Children’s Hospital, 860, Urushiyama, Aoi-ku, Shizuoka, 420-8660, Japan; Department of Cardiology, Shizuoka Children’s Hospital, 860, Urushiyama, Aoi-ku, Shizuoka, 420-8660, Japan; Department of Cardiology, Shizuoka Children’s Hospital, 860, Urushiyama, Aoi-ku, Shizuoka, 420-8660, Japan; Department of Cardiology, Shizuoka Children’s Hospital, 860, Urushiyama, Aoi-ku, Shizuoka, 420-8660, Japan

**Keywords:** Right heart failure, Arrhythmia, TPVI, ECMO, Sapien3, Case report

## Abstract

**Background:**

We report a case of successful transcatheter pulmonary valve implantation (TPVI) with elective extracorporeal membrane oxygenation (ECMO) support in a high-risk patient with complex congenital heart disease complicated by severe biventricular heart failure and ventricular tachycardia (VT).

**Case summary:**

A 55-year-old woman with a history of tetralogy of Fallot underwent surgical repair at the age of 3 years. At 50 years of age, the patient underwent bioprosthetic pulmonary valve replacement because of severe pulmonary regurgitation. Two years later, valve thrombosis required valve replacement, followed by infective endocarditis, leading to progressive pulmonary valve stenosis and regurgitation. This results in right ventricular dilation, biventricular failure, and recurrent VT. Given its high surgical risk, TPVI was selected. Owing to the impaired cardiac function and arrhythmia risk, elective ECMO support was planned to prevent intraoperative haemodynamic collapse. On the day of the procedure, ECMO was initiated in the hybrid OR, and TPVI was successfully performed using a 26 mm SAPIEN 3 valve. ECMO provided stable haemodynamics throughout the procedure, and the patient was successfully weaned postoperatively in the hybrid room. The patient had no complications and was discharged on postoperative Day 6.

**Discussion:**

Reports on TPVI with elective ECMO support are limited. This case suggests that in high-risk patients with severe heart failure and arrhythmias due to pulmonary valve dysfunction, elective ECMO can provide haemodynamic stability and allow TPVI to be safely performed with favourable outcomes.

Learning points

- Transcatheter pulmonary valve implantation (TPVI) offers a less invasive alternative to surgical pulmonary valve replacement and can be performed safely even in high-risk patients.
- In patients with biventricular heart failure and ventricular arrhythmias at high risk of intraoperative haemodynamic collapse, elective extracorporeal membrane oxygenation (ECMO) support allows safe completion of TPVI.
- Careful multidisciplinary planning and pre-emptive use of mechanical circulatory support can improve procedural safety in complex congenital heart disease interventions.

## Introduction

Recentry, transcatheter pulmonary valve implantation (TPVI) has been increasingly used as a less invasive treatment for pulmonary stenosis (PS) and pulmonary regurgitation (PR) in patients after right ventricular outflow tract reconstruction, such as those with tetralogy of Fallot (TOF).^1,2^ Some patients postoperatively present with severe heart failure and arrhythmias, however, there are currently no established treatment guidelines for such high-risk cases. We describe a case of successful TPVI performed with elective extracorporeal membrane oxygenation (ECMO) support in a high-risk patient.

## Summary figure

**Figure:**
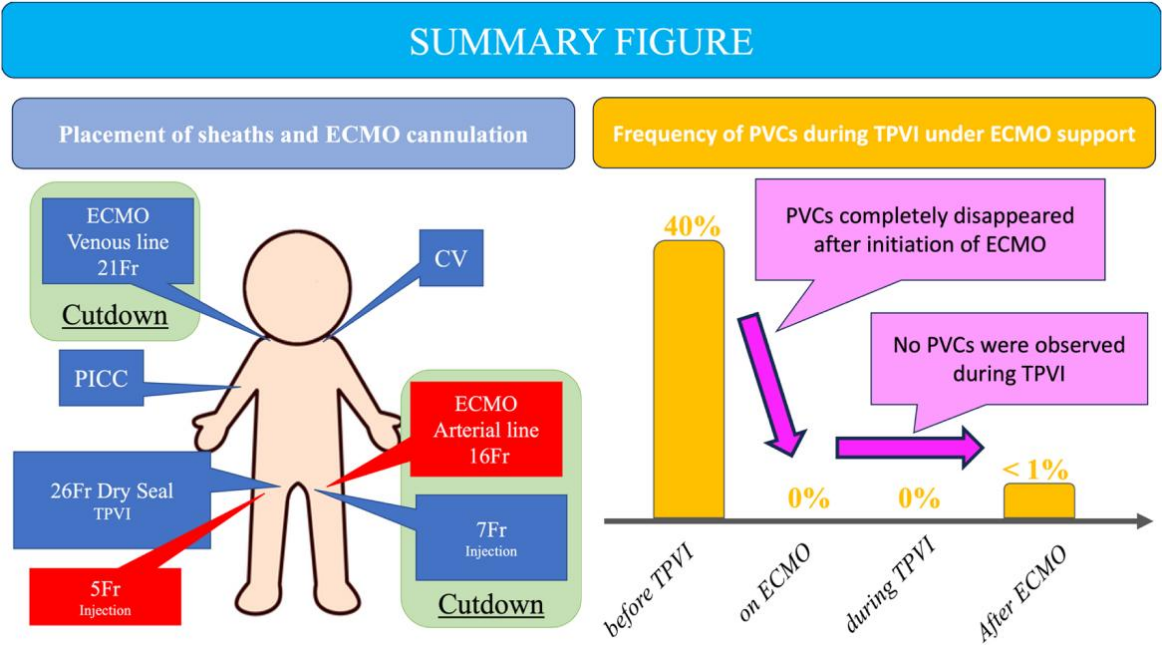


## Case presentation

**Figure 1:**
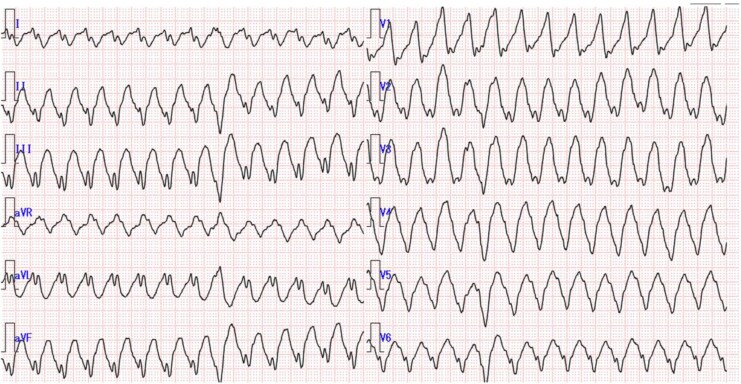
Twelve-lead electrocardiogram during ventricular tachycardia. Leads II, III, and aVF show an upward axis, indicating a ventricular tachycardia originating from the inferior wall.

The patient was a 55-year-old woman with a history of TOF repair at the age of 3 years. At the age of 50 years, she underwent pulmonary valve replacement (PVR) with a 25-mm INSPIRIS RESILIA^TM^ bioprosthetic valve (Edwards Lifesciences, Irvine, CA, USA) for severe PR. Two years later, replacement was required with a 27-mm SJM Epic valve (St. Jude Medical, St. Paul, MN, USA) because of valve thrombosis. Postoperatively, the patient developed mediastinitis and subsequently infective endocarditis. Over time, her pulmonary valve regurgitation worsened, and right ventricular dilation became apparent. Cardiac magnetic resonance imaging (cMRI) revealed a cardiac index (CI) of 1.6 L/min/m², left ventricular ejection fraction (LVEF) of 31%, right ventricular ejection fraction (RVEF) of 18%, right ventricular end-diastolic volume index (RVEDVI) of 245 mL/m², right ventricular end-systolic volume index (RVESVI) of 200 mL/m², and a PR fraction of 42%. In the most recent cardiac catheterization, the central venous pressure was measured at 8 mmHg, the mean pulmonary artery pressure ranged from 14 to 16 mmHg, the pulmonary artery wedge pressure was 10 mmHg, and the pressure gradient across the pulmonary valve was 20 mmHg. At the age of 55 years, Holter ECG revealed premature ventricular contractions (PVCs), accounting for 39% of the total heartbeats, and episodes of sustained ventricular tachycardia (VT) were also observed (*Figure 1*) while receving oral amiodarone. Although implantation of an implantable cardioverter-defibrillator was considered, haemodynamic stabilization was prioritized. The patient was referred to our hospital for PVR due to severe biventricular heart failure and arrhythmias.

**Figure 2:**
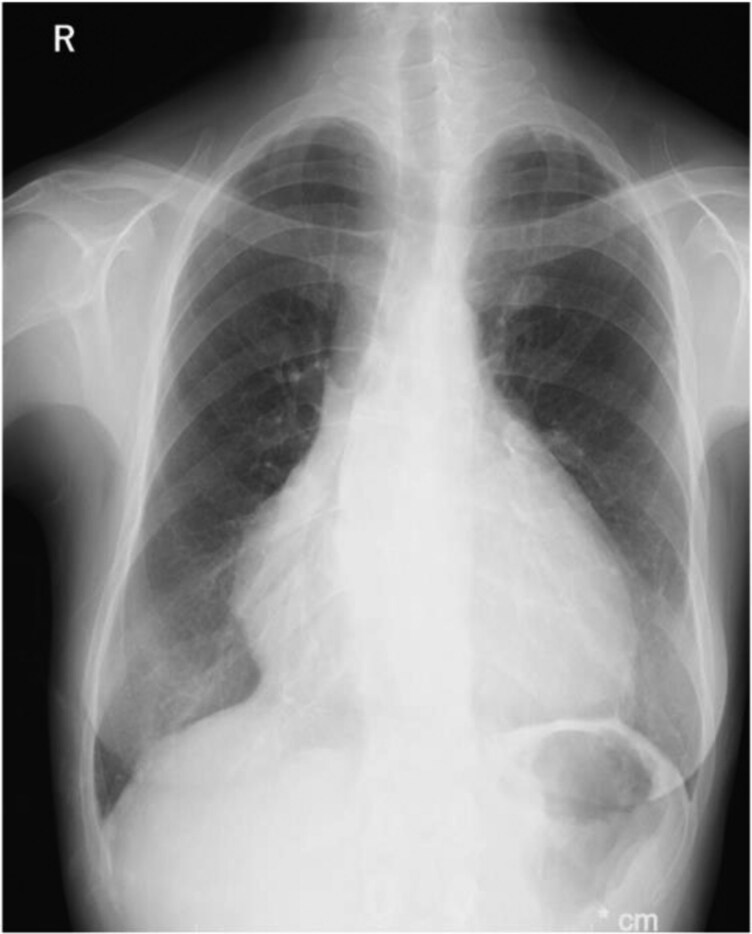
Chest radiograph showing cardiomegaly with a cardiothoracic ratio of 63.5%.

**Figure 3:**
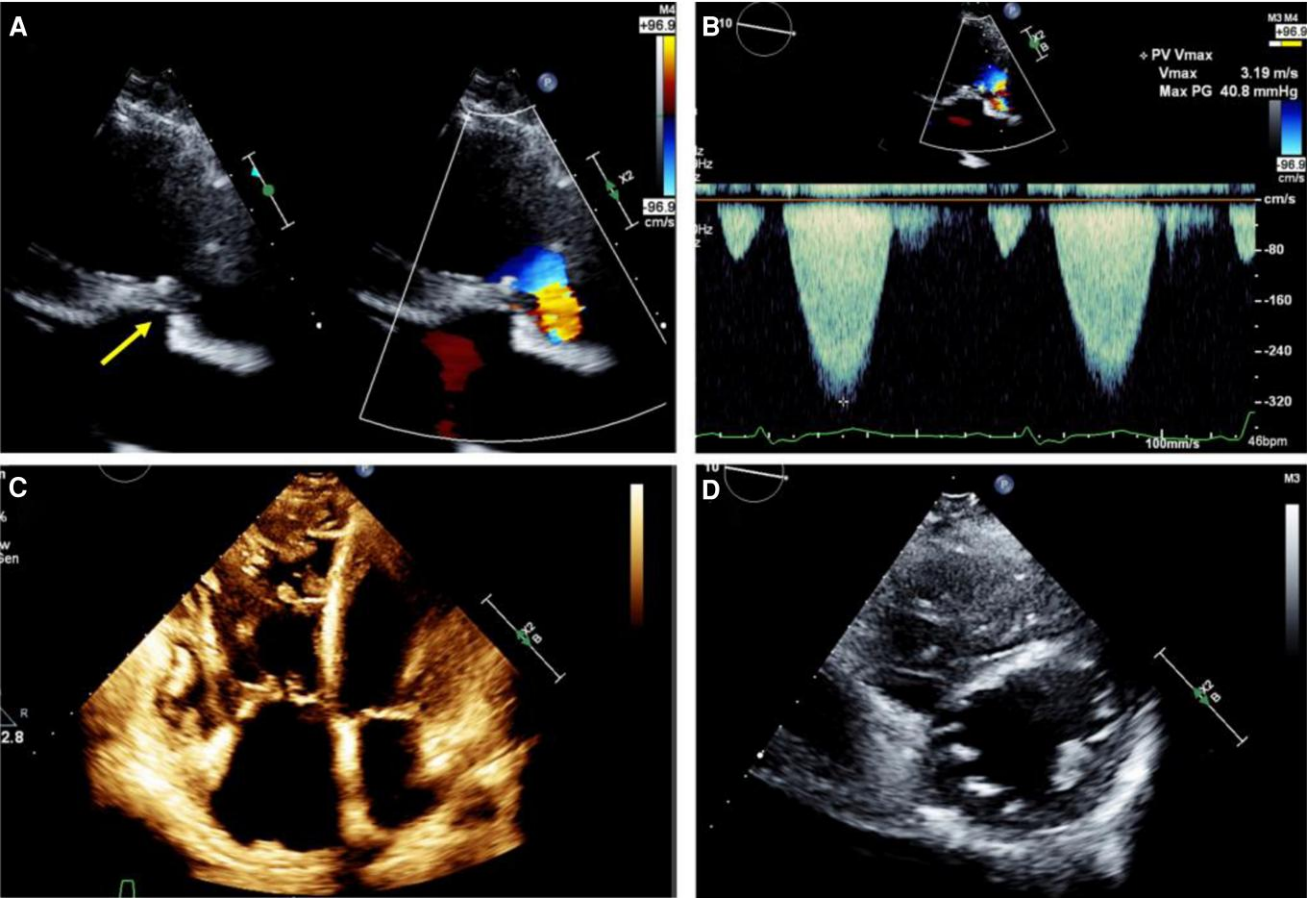
Transthoracic echocardiographic images. Two-dimensional echocardiographic image of the pulmonary artery stenosis site (arrow) (*A*) and Doppler measurement showing a peak velocity of 3.2 m/s and peak pressure gradient of 41 mmHg at the stenotic site (*B*). Apical four-chamber view and short-axis view prior to transcatheter pulmonary valve implantation demonstrating biventricular dysfunction with right ventricular pressure and volume overload (C and *D*).

The patient presented with easy fatigability, daily arrhythmias, and decreased appetite. Auscultation revealed a systolic murmur graded Levine 2/6 at the left sternal border of the second intercostal space, and a diastolic murmur graded Levine 1/6 at the left sternal border of the third intercostal space. Laboratory results showed elevated BNP (1521 pg/mL), BUN 25 mg/dL, Creatinine 2.0 mg/dL, and eGFR 21.5 mL/min/1.73m², indicating renal dysfunction. The chest X-ray showed a cardiothoracic ratio (CTR) of 63.5% (*Figure 2*). Transthoracic echocardiography revealed a PS velocity of 3.2 m/s with a peak pressure gradient of 41 mmHg with the presence of end-diastolic forward flow in the pulmonary artery (*Figure 3*, Supplementary material online, *Video S1*), indicating progression compared with data from the previous hospital. Additionally, LVEF was 41%, RV fractional area change was 22%, and RV tei index was 0.47 (see Supplementary material online, *Video S2*, *S3*).

Given the severe biventricular heart failure, frequent ventricular arrhythmias, renal dysfunction, and history of multiple thoracotomies and mediastinitis, the risk of open heart surgery under cardiopulmonary bypass is considered extremely high. Therefore, TPVI was selected as the primary treatment option. The patient exhibited frequent PVCs and VT under normal conditions, and it was anticipated that catheter manipulation during TPVI could readily lead to haemodynamic collapse. Therefore, ECMO support was selected to ensure stable haemodynamics during the intervention. A multidisciplinary conference was held to review the procedure, including sheath and cannulation site placement and intraoperative ECMO positioning. A peripherally inserted central catheter was inserted the day before the catheter intervention, and administration of catecholamines and amiodarone was initiated. Subsequently, the peak velocity across the pulmonary stenosis increased to 3.5 m/s following catecholamine administration. After entering the catheterization laboratory, the patient was intubated by an anaesthesiologist. A 21-Fr venous drainage cannula was inserted into the right internal jugular vein, and a 16-Fr arterial return cannula was placed in the left femoral artery to establish ECMO support. After ECMO was initiated, the PVCs completely disappeared. The ECMO was set to a perfusion index (PI) of 1.0 L/min/m², which was temporarily increased to 2.0 L/min/m² during critical stages such as pre-dilation and valve deployment.

**Figure 4:**
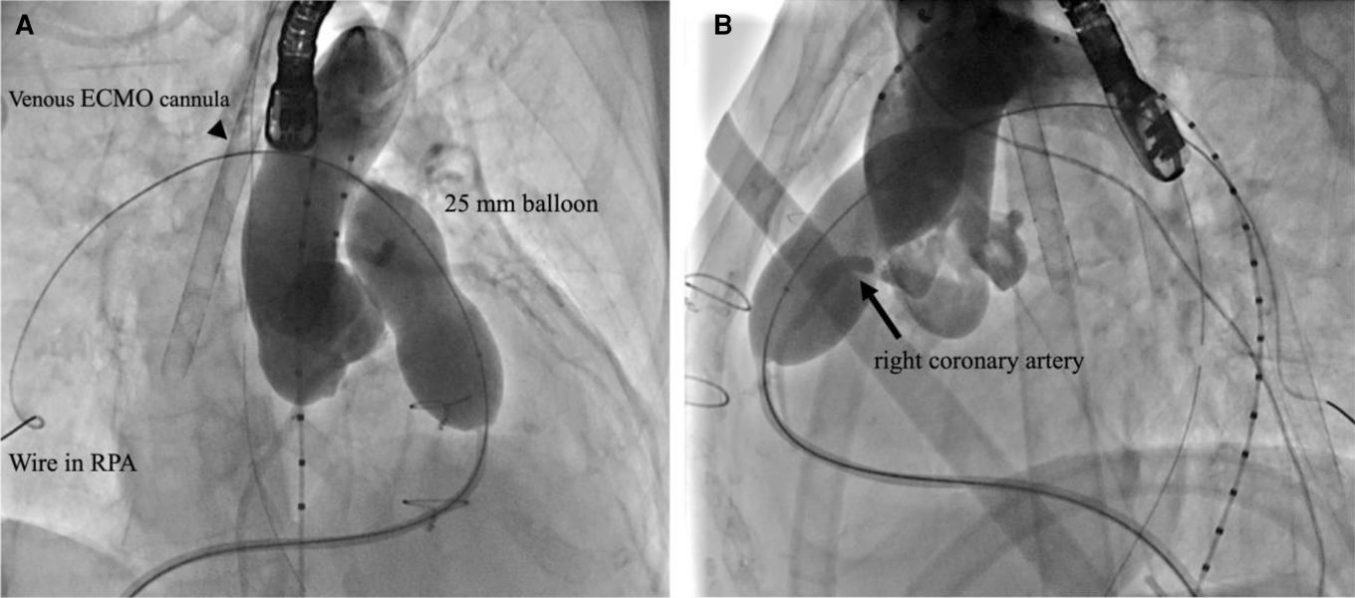
Cranial and lateral fluoroscopy views. Dilation of the pulmonary artery stenosis with a 25 mm balloon and aortography during the dilation without interference to the coronary arteries. (A and *B*) ECMO, extracorporeal membrane oxygenation; RPA, right pulmonary artery.

**Figure 5:**
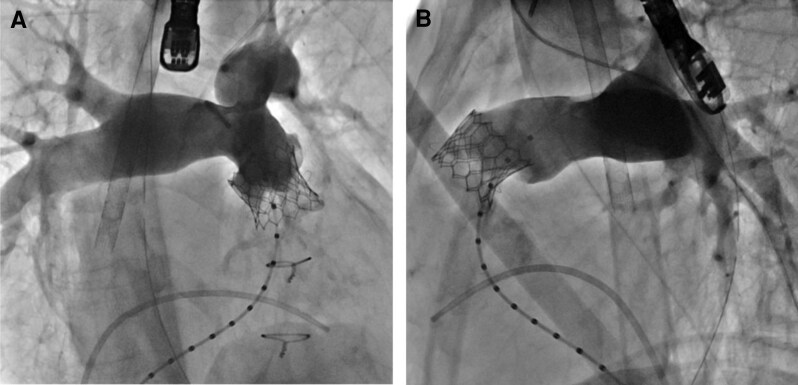
Fluoroscopy cranial and lateral views of pulmonary arteriography after a 26 mm Sapien3 implantation (*A* and *B*). No pulmonary insufficiency or gradient was observed.

**Figure 6:**
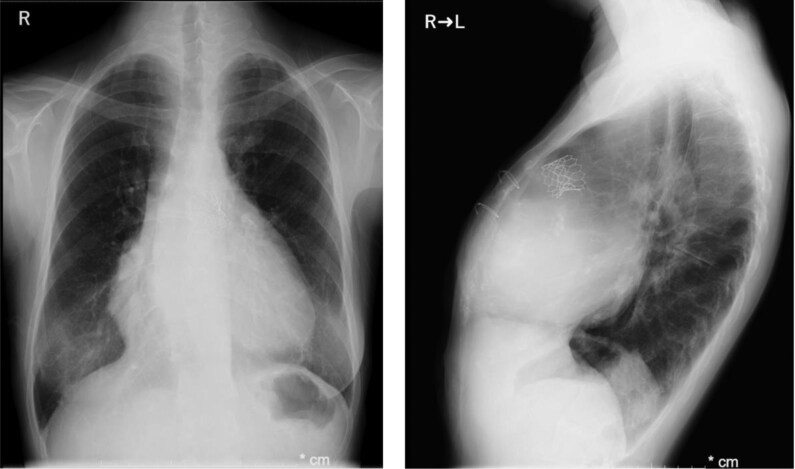
Postoperative Day 4 chest radiographs (frontal and lateral views) showing a cardiothoracic ratio of 62.6%.

**Figure 7:**
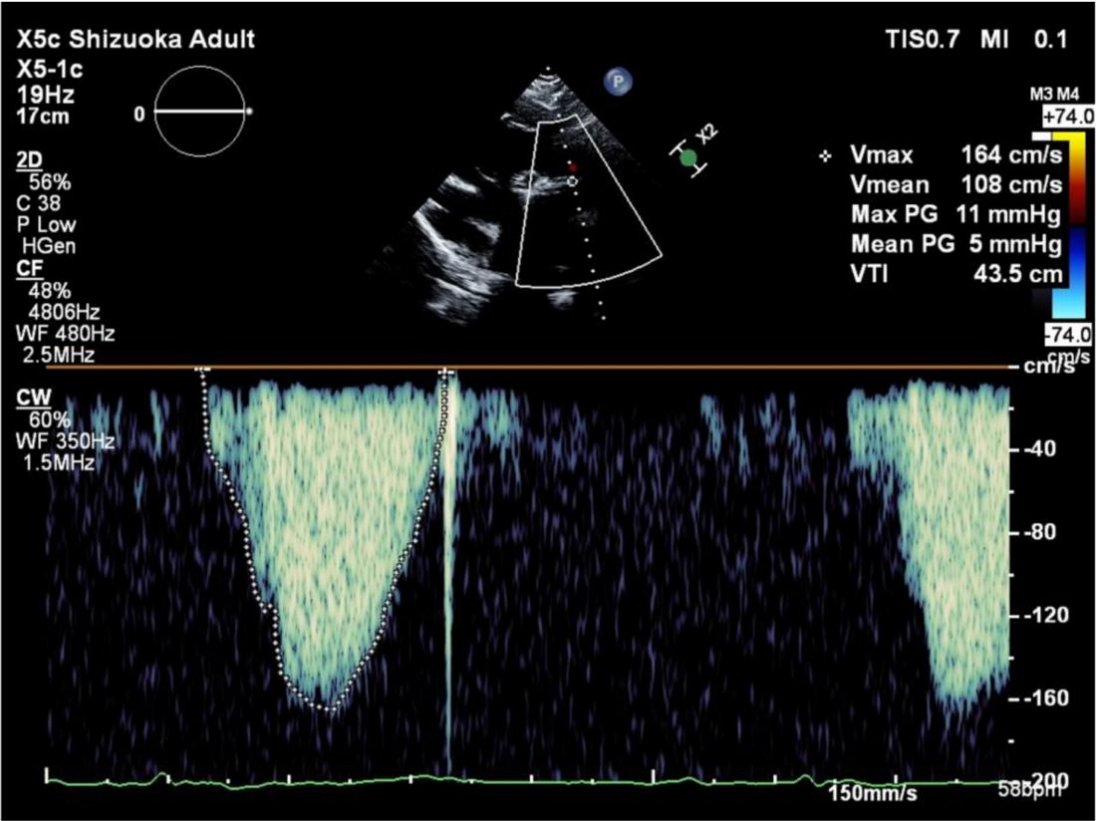
Transthoracic echocardiography on postoperative Day 5 confirming good mobility of the implanted valve and improvement of pulmonary stenosis (PS) and pulmonary regurgitation (PR), with no evidence of end-diastolic forward flow.

A 7-Fr balloon wedge-pressure catheter was placed in the right pulmonary artery, and a Lunderquist wire (Cook Medical, Inc., Bloomington, IN, USA) was positioned at the same site. A 25-mm Edwards balloon (Edwards Lifesciences, Irvine, CA, USA) was used to predilate the pulmonary valve at six atmospheres. During this step, aortic angiography was performed, and no coronary compression was observed (*Figure 4*). The sheath was replaced with a 26-Fr GORE DrySeal Flex Introducer Sheath (WL GORE, Newark, DE, USA). A 26 mm Sapien3 valve (Edwards Lifesciences, Irvine, CA, USA) was selected and deployed (*Figure 5*). Postprocedure, transoesophageal echocardiography confirmed the absence of PR, and the pressure gradient across the valve decreased from 20 to 0 mmHg under ECMO support with a PI of 1.0. Haemodynamics remained stable throughout the procedure, and no arrhythmias were observed. ECMO was performed in the catheterization laboratory, and the patient was extubated after sheath removal. Neurological assessment revealed no abnormalities, and the patient was transferred to the ICU. Catecholamine support was discontinued the following day. On postoperative Day 4, the chest X-ray showed a CTR of 62.6% (*Figure 6*). On postoperative Day 5, echocardiography confirmed mobility of the implanted valve and demonstrated improvement in PS and PR (*Figure 7*, Supplementary material online, *Video S4*). Holter ECG on postoperative Day 4 showed that the PVC frequency had decreased to less than 1%. The patient’s overall condition improved, and she was discharged on postoperative Day 6.

One month postoperatively, the patient’s BNP level decreased to 314 pg/mL and NYHA class improved from class Ⅲ preoperatively to class Ⅱ. A follow-up cMRI 2 months after the procedure showed a CI of 2.0 L/min/m², LVEF 35%, RVEF 26%, RVEDVI 182 mL/m², RVESVI 134 mL/m², and PR fraction 8%, indicating significant improvement in both right ventricular volume and function.

## Discussion

Due to severe PS and PR, the patient experienced decreased cardiac output, leading to severe biventricular heart failure and the potential for life-threatening arrhythmias such as VT. Compared to surgical PVR, TPVI is reported to be less invasive, associated with fewer procedure-related complications, and allows for a shorter hospital stay.^3^ Therefore, TPVI was considered a more favourable option in this case. However, owing to the presence of biventricular heart failure and the high likelihood of haemodynamic collapse in the event of intraoperative VT, TPVI with elective ECMO support is the most appropriate approach. Although the patient did not have hypoxaemia as an indication for ECMO, they presented with biventricular failure, primarily right-sided heart failure. Therefore, VA-ECMO was selected over VV-ECMO or Impella (Abiomed, Danvers, MA, USA). Several reports have suggested that catheter-based interventions with elective ECMO support are useful in high-risk cases.^4,5^ Sabbak *et al*.^6^ reported a case in which a 24-year-old patient who had previously undergone Rastelli repair underwent TPVI under emergency ECMO support. In their case, as in our case, the patient had biventricular failure and was considered to be at a high risk for surgical intervention; thus, TPVI was selected. However, during angiography in the catheterization laboratory, the patient experienced a cardiac arrest and required emergency ECMO. Following ECMO initiation, the patient’s condition stabilized, and TPVI was successfully performed as planned. Haemodynamics improved after the procedure, and ECMO was weaned off on the same day, as in our case. These examples suggest that in high-risk patients, the benefits of performing TPVI under elective ECMO support may outweigh the risks. In our case, a preoperative multidisciplinary conference was held with cardiovascular surgeons, anaesthesiologists, clinical engineers, and nurses to discuss vascular access, cannula size, procedural plan, and equipment setup. Fortunately, the procedure proceeded smoothly without major complications after admission to the catheterization laboratory. With VA-ECMO support, we avoided the occurrence of VT triggered by an increased afterload on the dysfunctional right ventricle during balloon dilation and valve deployment, and treatment was safely completed. The smooth course of the procedure minimized patient burden and, with improvement in haemodynamics, ECMO was successfully discontinued without complications shortly thereafter.

The successful outcome in this patient suggests that even in very high-risk cases, TPVI can be safely performed with hemodynamic support provided by elective ECMO.

## Conclusion

This case report describes a case of TPVI performed with elective ECMO support in a high-risk patient. In cases complicated by biventricular heart failure and ventricular arrhythmias due to PR and PS, where there is a high risk of intraoperative haemodynamic collapse, elective ECMO support may reduce circulatory instability and allow safe procedures.

## Lead author biography


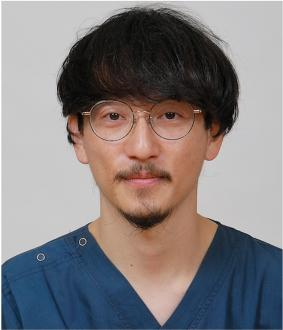


Dr. Hidehiro Mori is a medical doctor at Shizuoka Children's Hospital, specializing in catheter interventions. After completing his residency at Osaka City General Hospital, he developed both clinical and research interests in congenital heart disease.

## Supplementary Material

ytag044_Supplementary_Data

## Data Availability

The data underlying this study are available for this article.
